# Supplementary material for: Job satisfaction of clinical pharmacists and clinical pharmacy activities implemented at Ho Chi Minh city, Vietnam
Source: PLoS One. 2021 Jan 22;16(1):e0245537. doi: 10.1371/journal.pone.0245537 (PMC7822262; doi:10.1371/journal.pone.0245537)
Supplement: S1 Table — (DOCX) [file pone.0245537.s001.docx]

**S1 Table.** Factor analysis result and clinical pharmacists’ satisfaction on each item (N = 197)

| **Items** | **Factor loading** | **Mean**  **(SD)** | **No.of CPs satisfied**  **[n (%)]** | **Factors** |
| --- | --- | --- | --- | --- |
| Hospital executives are capable of handling, administering and solving work effectively | 0.686 | 4.03 (0.64) | 168 (85.3) | **Executives and internal regulation scale** |
| Hospital executives encourage and praise you when you perform excellently at work | 0.605 | 3.83 (0.78) | 146 (74.1) |  |
| Hospital executives have fair treatment for everyone | 0.688 | 3.92 (0.71) | 159 (80.7) |  |
| Hospital executives respect all staff opinions | 0.643 | 3.96 (0.68) | 159 (80.7) |  |
| Hospital executives has paid attention to clinical pharmacy services | 0.584 | 4.05 (0.69) | 167 (84.8) |  |
| Hospital executives assigns jobs in accordance with the staff's training expertise | 0.694 | 3.97 (0.63) | 170 (86.3) |  |
| The Head of the Pharmacy department supports you in work | 0.753 | 4.09 (0.71) | 175 (88.8) |  |
| The Head of the Pharmacy department has paid attention and invested in building strategies to promote clinical pharmacy activities in the hospital | 0.717 | 4.05 (0.69) | 171 (86.8) |  |
| The hospital's internal regulations and rules are clear, realistic and public | 0.517 | 3.92 (0.63) | 166 (84.3) |  |
| Your salary matches up to your competence and contribution | 0.797 | 3.32 (0.96) | 94 (47.7) | **Income** |
| The hospital has appropriate policies in distributing benefits to employees | 0.712 | 3.67 (0.75) | 134 (68) |  |
| Occupational allowance and hazardous allowance matches the dedication | 0.806 | 3.62 (0.82) | 131 (66.5) |  |
| Bonus and extra income matches the dedication | 0.851 | 3.49 (0.88) | 116 (58.9) |  |
| Bonus and extra income are distributed fairly, which encourages employees to work harder | 0.732 | 3.58 (0.78) | 124 (62.9) |  |
| You are offered vacations regularly | 0.808 | 3.96 (0.69) | 169 (85.8) | **Benefits** |
| Hospital organizes sports competitions and arts programs for employees regularly | 0.768 | 4.02 (0.58) | 177 (89.8) |  |
| The hospital union is active | 0.747 | 3.98 (0.7) | 172 (87.3) |  |
| The hospital has policies supporting staff to improve their qualifications (degrees). | 0.687 | 3.89 (0.74) | 159 (80.7) | **Trainings, promotion opportunities** |
| The hospital creates favorable conditions for staff to participate in short-term training courses to improve soft skills (communication skills, informatics,...) | 0.706 | 4.03 (0.68) | 169 (85.8) |  |
| The hospital organizes continuing medical education courses for members on clinical pharmacy, drug information,.... | 0.592 | 3.67 (0.86) | 140 (71.1) |  |
| The hospital facilitates employees to participate in courses on hospital management skills (updating pharmaceutical management documents, quality management and patient safety, human resource management, information management. ..) | 0.686 | 3.88 (0.68) | 154 (78.2) |  |
| You have promotion opportunities during the working process for your effort | 0.535 | 3.66 (0.81) | 131 (66.5) |  |
| Hospital’s promotion policy is transparent and reasonable | 0.623 | 3.65 (0.78) | 126 (64) |  |
| Your current job is in accordance with the expertise in which you are trained | 0.720 | 4.01 (0.55) | 174 (88.3) | **Job characteristic** |
| You have the opportunity to develop your capacity at work. | 0.696 | 3.95 (0.57) | 167 (84.8) |  |
| The workload is assigned appropriately | 0.585 | 3.76 (0.68) | 143 (72.6) |  |
| Your current job is interesting | 0.782 | 3.78 (0.68) | 140 (71.1) |  |
| The specialized job meets your personal aspirations | 0.750 | 3.89 (0.68) | 159 (80.7) |  |

**S1 Table.** Factor analysis result and clinical pharmacists’ satisfaction on each item (N = 197) (continued)

| **Items** | **Factor loading** | **Mean**  **(SD)** | **No.of CPs satisfied**  **[n (%)]** | **Factors** |
| --- | --- | --- | --- | --- |
| Your working is equipped with sufficient sources of clinical pharmacy-related materials | 0.629 | 3.76 (0.81) | 140 (71.1) | **Working condition** |
| You are satisfied with the facilities at your workplace (The office is spacious, clean, and airy) | 0.700 | 3.77 (0.74) | 143 (72.6) |  |
| The hospital has enough equipment for your professional work. (Office equipments, desks and chair,... are adequate; old and outdated equipments will be replaced promptly) | 0.794 | 3.93 (0.68) | 164 (83.2) |  |
| The learning environment (library, reading room, information search, internet access) creates conditions for you to update your knowledge and to improve your qualifications | 0.695 | 3.81 (0.69) | 152 (77.2) |  |
| You are equipped with adequate and new protective gears (clothing, mask, gloves,...) and use is unrestricted. | 0.622 | 4.01 (0.59) | 177 (89.8) |  |
| You are having a safe working environment | 0.604 | 4.02 (0.54) | 179 (90.9) |  |
| Your pharmacy colleagues have good cooperation with you | 0.601 | 4.09 (0.59) | 178 (90.4) | **Inter & Intra professional relationship** |
| You have an affable relationship with your pharmacy colleagues | 0.720 | 4.19 (0.52) | 188 (95.4) |  |
| You have a good professional collaboration with physicians | 0.659 | 3.98 (0.57) | 165 (88.3) |  |
| You have a good professional collaboration with nurses | 0.667 | 3.97 (0.57) | 165 (88.3) |  |
| Coworkers support each other in life | 0.657 | 4.05 (0.57) | 174 (88.3) |  |
| Coworkers are willing to share experiences and support each other in work | 0.691 | 4.14 (0.6) | 178 (90.4) |  |
| Overall satisfaction level about the hospital | 0.867 | 3.85 (0.69) | 151 (76.6) | **Overall job satisfaction** |
| Self-assessment of the level of workload completion at the hospital | 0.867 | 3.84 (0.58) | 146 (74.1) |  |
| Overall satisfaction level on your current job | 0.845 | 3.75 (0.65) | 135 (68.5) |  |
| The working environment in hospital departments is democratic. | - | *9 items eliminated because*  *factor loadings <0.5* | | |
| The internal spending regulations are fair, reasonable and public | - |  |  |  |
| Hospital ensures the full payment of social insurance, health insurance, periodic health check, other forms of sickness and maternity support | - |  |  |  |
| Hospital publishes all the leadership standards | - |  |  |  |
| Hospital appointes leadership position democratically and equally. | - |  |  |  |
| The hospital ensures security and order for you to fully focus on working | - |  |  |  |
| Patients and their families have a respectful and collaborative attitude with you during treatment process | - |  |  |  |
| There are duty rooms for you | - |  |  |  |
| Your duty time is arranged reasonably | - |  |  |  |
