# Supplementary material for: Job satisfaction of clinical pharmacists and clinical pharmacy activities implemented at Ho Chi Minh city, Vietnam
Source: PLoS One. 2021 Jan 22;16(1):e0245537. doi: 10.1371/journal.pone.0245537 (PMC7822262; doi:10.1371/journal.pone.0245537)
Supplement: S1 File — (DOCX) [file pone.0245537.s002.docx]

## QUESTIONNAIRE (VIETNAMESE VERSION)

## PHẦN I. THÔNG TIN CÁ NHÂN

**Xin vui lòng hoàn thành các thông tin dưới đây bằng cách khoanh tròn vào chữ số trước câu trả lời được chọn hoặc điền vào chỗ trống:**

1. **Giới tính:** 1. Nam 2. Nữ 3. Khác
2. **Năm sinh:**
3. **Tình trạng hôn nhân:**  1. Độc thân 2. Đã kết hôn 3. Khác
4. **Tên bệnh viện đang công tác**:
5. **Trình độ chuyên môn (CÓ THỂ CHỌN NHIỀU ĐÁP ÁN):**

| 1. Đại học | 4. Chuyên khoa I |
| --- | --- |
| 2. Thạc sĩ | 5. Chuyên khoa II |
| 3. Tiến sĩ |  |

1. **Vị trí công tác hiện tại:**

| 1. Lãnh đạo bệnh viện | 4. Nhân viên biên chế/hợp đồng dài hạn |
| --- | --- |
| 2. Trưởng Khoa/phòng/trung tâm | 5. Hợp đồng ngắn hạn |
| 3. Phó khoa/phòng | 6. Khác: |

1. **Anh/Chị có được phân công kiêm nhiệm nhiều công việc không?**

| 1. Không kiêm nhiệm |
| --- |
| 2. Kiêm nhiệm 2 công việc |
| 3. Kiêm nhiệm từ 3 công việc trở lên |

1. **Số giờ phụ trách công tác dược lâm sàng trong 1 tuần:** giờ/tuần
2. **Số giờ phụ trách các công tác dược truyền thống trong 1 tuần:** giờ/tuần
3. **Số giờ tham gia đi buồng bệnh cùng bác sĩ/điều dưỡng:** giờ/tuần
4. **Thu nhập hằng tháng (triệu đồng/tháng)**

| 1. ≤10 |  | 2. >10 |  |
| --- | --- | --- | --- |

## PHẦN II. CÁC HOẠT ĐỘNG DƯỢC LÂM SÀNG

**Xin vui lòng trả lời các câu hỏi dưới đây bằng cách khoanh tròn vào chữ số trước câu trả lời được chọn**

1. **Trong các nhiệm vụ chung của Dược sĩ lâm sàng, Anh/Chị có tham gia vào hoạt động nào dưới đây?**

**(CÓ THẾ CHỌN NHIỀU ĐÁP ÁN)**

| 1. Tham gia phân tích, đánh giá tình hình sử dụng thuốc |
| --- |
| 2. Tham gia tư vấn trong quá trình xây dựng danh mục thuốc của đơn vị, đưa ra ý kiến thuốc nào nên đưa vào hoặc bỏ ra khỏi danh mục thuốc để bảo đảm mục tiêu sử dụng thuốc an toàn, hợp lý và hiệu quả |
| 3. Tham gia xây dựng các quy trình chuyên môn liên quan đến sử dụng thuốc: quy trình pha chế thuốc, hướng dẫn điều trị, quy trình kỹ thuật của bệnh viện |
| 4. Tham gia xây dựng quy trình giám sát sử dụng đối với các thuốc trong danh mục (bao gồm các thuốc có khoảng điều trị hẹp, nhiều tác dụng phụ nghiêm trọng, kháng sinh, thuốc cần pha truyền đặc biệt (chuyên khoa nhi, ung bướu), thuốc cần điều kiện bảo quản đặc biệt) do Giám đốc bệnh viện ban hành trên cơ sở được tư vấn của Hội đồng Thuốc và Điều trị |
| 5. Hướng dẫn và giám sát việc sử dụng thuốc trong bệnh viện |
| 6. Thông tin thuốc cho người bệnh và cán bộ y tế: dược sĩ lâm sàng cập nhật thông tin sử dụng thuốc, thông tin về thuốc mới, thông tin cảnh giác dược gửi đến cán bộ y tế và đến người bệnh bằng nhiều hình thức khác nhau như: trực tiếp, văn bản, bảng tin bệnh viện, thư điện tử, tranh ảnh, tờ hướng dẫn, trang thông tin điện tử |
| 7. Tập huấn, đào tạo về dược lâm sàng: dược sĩ lâm sàng lập kế hoạch, chuẩn bị tài liệu, cập nhật kiến thức sử dụng thuốc cho bác sĩ, dược sĩ, điều dưỡng viên, kỹ thuật viên, hộ sinh viên của đơn vị mình. Kế hoạch và nội dung phải được Giám đốc bệnh viện phê duyệt; |
| 8. Báo cáo định kỳ hằng tháng, hằng quý, hằng năm và báo cáo đột xuất theo yêu cầu của Ban Giám đốc, Hội đồng Thuốc và Điều trị: Dược sĩ lâm sàng báo cáo công tác sử dụng thuốc trong buổi họp của Hội đồng Thuốc và Điều trị hoặc buổi giao ban của đơn vị, có ý kiến trong các trường hợp sử dụng thuốc chưa phù hợp |
| 9. Theo dõi, giám sát phản ứng có hại của thuốc (ADR) và là đầu mối báo cáo các phản ứng có hại của thuốc tại đơn vị theo quy định hiện hành |
| 10. Tham gia các hoạt động, công trình nghiên cứu khoa học, đặc biệt là các nghiên cứu liên quan đến vấn đề sử dụng thuốc an toàn - hợp lý, vấn đề cải tiến chất lượng và nâng cao hiệu quả công tác dược lâm sàng, nghiên cứu sử dụng thuốc trên lâm sàng |
| 11. Tham gia hội chẩn chuyên môn về thuốc, đặc biệt trong các trường hợp bệnh nặng, bệnh cần dùng thuốc đặc biệt, người bệnh bị nhiễm vi sinh vật kháng thuốc |
| 12. Tham gia bình ca lâm sàng định kỳ tại khoa lâm sàng, tại bệnh viện |
| 13. Giám sát chặt chẽ việc tuân thủ quy trình sử dụng thuốc đã được Hội đồng Thuốc và Điều trị thông qua và Giám đốc bệnh viện phê duyệt |

14. Tham gia xây dựng và thực hiện quy trình giám sát điều trị thông qua theo dõi nồng độ thuốc trong máu (Therapeutic Drug Monitoring - TDM)

1. **Trong các nhiệm vụ tại khoa lâm sàng của Dược sĩ lâm sàng, Anh/Chị có tham gia vào hoạt động nào dưới đây?**

**(CÓ THẾ CHỌN NHIỀU ĐÁP ÁN)**

| 1. Khai thác thông tin của người bệnh (bao gồm cả khai thác thông tin trên bệnh án và tiến hành phỏng vấn trực tiếp người bệnh) về: tiền sử sử dụng thuốc; tóm tắt các dữ kiện lâm sàng và các kết quả cận lâm sàng đã có |
| --- |
| 2. Xem xét các thuốc được kê đơn cho người bệnh (trong quá trình đi buồng bệnh cùng với bác sĩ và xem xét y lệnh trong hồ sơ bệnh án, đơn thuốc) về: chỉ định, chống chỉ định, lựa chọn thuốc, dùng thuốc cho người bệnh (liều dùng, khoảng cách dùng, thời điểm dùng, đường dùng, dùng thuốc trên các đối tượng đặc biệt, thời gian dùng thuốc), các tương tác thuốc cần chú ý, phản ứng có hại của thuốc |
| 3. Hướng dẫn sử dụng thuốc cho điều dưỡng viên |
| 4. Phối hợp với bác sĩ điều trị để cung cấp thông tin tư vấn cho người bệnh về những điều cần lưu ý trong quá trình sử dụng thuốc. |

## PHẦN III. SỰ HÀI LÒNG TRONG CÔNG VIỆC

1. **Xin vui lòng cho biết mức độ đồng ý/hài lòng của Anh/Chị về các vấn đề sau, bằng cách khoanh tròn vào chữ số ứng với câu trả lời mà Anh/Chị cho là phù hợp nhất**

(Hãy cho điểm từ 1 đến 5 với 1 là “Rất không đồng ý/Rất không hài lòng” và 5 là “Rất đồng ý/Rất hài lòng”)

| **Các nội dung** | **Mức độ đồng ý/hài lòng** | | | | |
| --- | --- | --- | --- | --- | --- |
| 1. Lãnh đạo có năng lực xử lý, điều hành, giải quyết công việc hiệu quả | 1 | 2 | 3 | 4 | 5 |
| 1. Lãnh đạo bệnh viện có các buổi gặp mặt nhằm khích lệ nhân viên khi hoàn thành tốt nhiệm vụ, có tiến bộ trong công việc, quan tâm đến đời sống nhân viên | 1 | 2 | 3 | 4 | 5 |
| 1. Lãnh đạo bệnh viện đối xử công bằng với tất cả mọi người trong bệnh viện | 1 | 2 | 3 | 4 | 5 |
| 1. Lãnh đạo bệnh viện tôn trọng ý kiến nhân viên | 1 | 2 | 3 | 4 | 5 |
| 1. Lãnh đạo bệnh viện có chú trọng đến hoạt động dược lâm sàng tại bệnh viện | 1 | 2 | 3 | 4 | 5 |
| 1. Lãnh đạo phân công công việc phù hợp với chuyên môn đào tạo của nhân viên | 1 | 2 | 3 | 4 | 5 |
| 1. Trưởng khoa dược quan tâm và giúp đỡ Anh/Chị trong công việc | 1 | 2 | 3 | 4 | 5 |
| 1. Trưởng khoa dược có sự quan tâm và đầu tư xây dựng các chiến lược nhằm đẩy mạnh hoạt động dược lâm sàng tại bệnh viện | 1 | 2 | 3 | 4 | 5 |
| 1. Các đồng nghiệp khoa dược có sự hợp tác tốt với Anh/Chị trong công việc | 1 | 2 | 3 | 4 | 5 |
| 1. Quan hệ của Anh/Chị với đồng nghiệp khoa dược rất thân thiện | 1 | 2 | 3 | 4 | 5 |
| 1. Anh/Chị có mối quan hệ hợp tác tốt với các bác sĩ trong bệnh viện | 1 | 2 | 3 | 4 | 5 |
| 1. Anh/Chị có mối quan hệ hợp tác tốt với các điều dưỡng viên trong bệnh viện | 1 | 2 | 3 | 4 | 5 |
| 1. Đồng nghiệp quan tâm, giúp đỡ nhau trong cuộc sống | 1 | 2 | 3 | 4 | 5 |
| 1. Đồng nghiệp chia sẻ kinh nghiệm, giúp đỡ nhau trong công việc | 1 | 2 | 3 | 4 | 5 |
| 1. Bệnh viện có chính sách hỗ trợ nhân viên học tập nâng cao trình độ chuyên môn (bằng cấp) | 1 | 2 | 3 | 4 | 5 |
| 1. Bệnh viện có tạo điều kiện cho nhân viên tham gia những khóa tập huấn ngắn hạn nâng cao kỹ năng mềm (kỹ năng giao tiếp, tin học,…) | 1 | 2 | 3 | 4 | 5 |
| 1. Bệnh viện có tổ chức các khóa đào tạo liên tục cho nhân viên về dược lâm sàng, thông tin thuốc,… | 1 | 2 | 3 | 4 | 5 |
| 1. Bệnh viện tạo điều kiện cho nhân viên tham gia các khóa học về kỹ năng quản lý bệnh viện (cập nhật văn bản quản lý dược, quản lý chất lượng và an toàn cho bệnh nhân, quản lý nguồn nhân lực, quản lý thông tin,…) | 1 | 2 | 3 | 4 | 5 |
| 1. Anh/Chị có nhiều cơ hội thăng tiến trong quá trình làm việc khi nỗ lực làm việc | 1 | 2 | 3 | 4 | 5 |
| 1. Bệnh viện có chính sách thăng tiến rõ ràng | 1 | 2 | 3 | 4 | 5 |
| 1. Công khai các tiêu chuẩn cho các chức danh lãnh đạo | 1 | 2 | 3 | 4 | 5 |
| 1. Bổ nhiệm các chức danh lãnh đạo dân chủ, công bằng | 1 | 2 | 3 | 4 | 5 |
| 1. Mức lương tương xứng so với năng lực và cống hiến | 1 | 2 | 3 | 4 | 5 |
| 1. Bệnh viện có chính sách phân chia nguồn phúc lợi cho nhân viên một cách hợp lý | 1 | 2 | 3 | 4 | 5 |
| 1. Chế độ phụ cấp nghề và độc hại xứng đáng so với cống hiến | 1 | 2 | 3 | 4 | 5 |
| 1. Thưởng và thu nhập tăng thêm ABC xứng đáng so với cống hiến | 1 | 2 | 3 | 4 | 5 |
| 1. Cách phân chia thu nhập tăng thêm công bằng, khuyến kích nhân viên làm việc tích cực | 1 | 2 | 3 | 4 | 5 |
| 1. Các quy định, quy chế làm việc nội bộ của bệnh viện rõ ràng, thực tế và công khai | 1 | 2 | 3 | 4 | 5 |
| 1. Môi trường làm việc tại khoa/phòng và bệnh viện dân chủ | 1 | 2 | 3 | 4 | 5 |
| 1. Quy chế chi tiêu nội bộ công bằng, hợp lý, công khai | 1 | 2 | 3 | 4 | 5 |
| 1. Bảo đảm đóng BHXH, BHYT, khám sức khỏe định kỳ và các hình thức hỗ trợ ốm đau, thai sản đầy đủ | 1 | 2 | 3 | 4 | 5 |
| 1. Tổ chức tham quan, nghỉ dưỡng đầy đủ | 1 | 2 | 3 | 4 | 5 |
| 1. Có phong trào thể thao, văn nghệ tích cực | 1 | 2 | 3 | 4 | 5 |
| 1. Công đoàn bệnh viện hoạt động tích cực | 1 | 2 | 3 | 4 | 5 |
| 1. Bệnh viện có đủ tài liệu phục vụ cho công việc chuyên môn của Anh/Chị | 1 | 2 | 3 | 4 | 5 |
| 1. Anh/Chị hài lòng với cơ sở vật chất nơi làm việc của mình. (Phòng làm việc khang trang, sạch sẽ, thoáng mát) | 1 | 2 | 3 | 4 | 5 |
| 1. Bệnh viện có đủ trang thiết bị phục vụ cho công việc chuyên môn của Anh/Chị. (Trang thiết bị văn phòng, bàn ghế làm việc… đầy đủ, các thiết bị cũ, lạc hậu được thay thế kịp thời) | 1 | 2 | 3 | 4 | 5 |
| 1. Môi trường học tập tạo điều kiện cho NVYT cập nhật kiến thức, nâng cao trình độ: thư viện, phòng đọc, tra cứu thông tin, truy cập internet | 1 | 2 | 3 | 4 | 5 |
| 1. Có trang bị bảo hộ của NVYT (quần áo, khẩu trang, găng tay…) đầy đủ, không bị cũ, nhàu nát, không bị hạn chế sử dụng | 1 | 2 | 3 | 4 | 5 |
| 1. Môi trường làm việc đảm bảo an toàn cho NVYT | 1 | 2 | 3 | 4 | 5 |
| 1. Bệnh viện bảo đảm an ninh, trật tự cho NVYT làm việc | 1 | 2 | 3 | 4 | 5 |
| 1. Người bệnh và người nhà có thái độ tôn trọng, hợp tác với NVYT trong quá trình điều trị | 1 | 2 | 3 | 4 | 5 |
| 1. Có bố trí phòng trực cho NVYT | 1 | 2 | 3 | 4 | 5 |
| 1. Phân chia thời gian trực và làm việc ngoài giờ hành chính hợp lý | 1 | 2 | 3 | 4 | 5 |
| 1. Công việc hiện tại phù hợp với chuyên môn mà Anh/Chị được đào tạo | 1 | 2 | 3 | 4 | 5 |
| 1. Anh/Chị có cơ hội phát huy được năng lực của mình trong công việc | 1 | 2 | 3 | 4 | 5 |
| 1. Khối lượng công việc được giao phù hợp | 1 | 2 | 3 | 4 | 5 |
| 1. Công việc hiện tại rất thú vị | 1 | 2 | 3 | 4 | 5 |
| 1. Công việc chuyên môn đáp ứng nguyện vọng bản thân | 1 | 2 | 3 | 4 | 5 |
| 1. Mức độ hài lòng nói chung về bệnh viện | 1 | 2 | 3 | 4 | 5 |
| 1. Tự đánh giá về mức độ hoàn thành công việc tại bệnh viện | 1 | 2 | 3 | 4 | 5 |
| 1. Mức độ hài lòng nói chung với công việc hiện tại | 1 | 2 | 3 | 4 | 5 |

## QUESTIONNAIRE (ENGLISH TRANSLATION)

## SECTION I. DEMOGRAPHIC AND WORK-RELATED INFORMATION

**Please complete all fields below**

1. **Gender:** 1. Male 2. Female 3. Other
2. **Year of birth:**
3. **Marital status:**  1. Single 2. Married 3. Other
4. **Name of working place**:
5. **Your academic degree (You can select multiple options):**

| 1. B. Pharm | 4. F.D.S. Pharm |
| --- | --- |
| 2. M. Pharm | 5. S.D.S. Pharm |
| 3. Ph.D. Pharm |  |

1. **Your current job position:**

| 1. Director of hospital | 4. Official employee |
| --- | --- |
| 2. Head of Pharmacy Department | 5. Short-term employee |
| 3. Deputy Head of Pharmacy Department | 6. On-probation employee |

1. **Number of duties that you are handling?**

| 1. Only 1 duty (clinical pharmacy) |
| --- |
| 2. 2 duties (clinical pharmacy + 1 traditional pharmacy duty) |
| 3. ≥ 3 duties (clinical pharmacy + ≥2 traditional pharmacy duties) |

1. **Number of hours spent on clinical pharmacy duty/week:** hour(s)/week
2. **Number of hours spent on traditional pharmacy duty/week:** hour(s)/week
3. **Number of hours spent on clinical ward rounds/week:** hour(s)/week
4. **Average income (USD/month)**

| 1. ≤433 |  | 2. >433 |  |
| --- | --- | --- | --- |

## SECTION II. CLINICAL PHARMACY TASKS

1. **Among 14 general clinical pharmacy tasks, which one(s) do you participate in? (You can select multiple options)**
2. Provide drug information for patients and medical employees
3. Participate in the analysis and evaluation of the drug use process
4. Provide guidelines and supervision for the use of drugs in the hospital
5. Monitor and report the adverse reactions of drugs at the unit
6. Trainings in clinical pharmacy
7. Participate in the periodic clinical case study in the hospital
8. Provide monthly, quarterly, annual and surprising reports about the drug use activities
9. Strictly supervise the compliance with the drug use process
10. Participate in developing the specialized processes related to the use of drugs
11. Participate in the scientific research activities and works
12. Participate in consultation during the process of developing the unit’s list of drugs
13. Participate in developing the process of supervising the use of listed drugs
14. Participate in specialized consultation about drugs
15. Participate in developing and carrying out the Therapeutic Drug Monitoring
16. **Among 4 clinical pharmacy tasks in the clinical wards, which one(s) do you participate in? (You can select multiple options)**

| 1. Examine the drugs prescribed for the patients |
| --- |
| 1. Provide drug instructions for nurses |
| 1. Cooperate with physicians to provide counseling for patients |
| 1. Exploit information from the patient (through clinical record and patient interview) |

## SECTION III. JOB SATISFACTION

1. **Please choose the number which corresponds the most with your level of satisfaction/agreement**

(1 to 5 corresponds with “Strongly dissatisfied/Strongly disagree” to “Strongly satisfied/Strongly agree”)

| **Item** | **Level of satisfaction/agreement** | | | | |
| --- | --- | --- | --- | --- | --- |
| 1. Hospital executives are capable of handling, administering and solving work effectively | 1 | 2 | 3 | 4 | 5 |
| 1. Hospital executives encourage and praise you when you perform excellently at work | 1 | 2 | 3 | 4 | 5 |
| 1. Hospital executives have fair treatment for everyone | 1 | 2 | 3 | 4 | 5 |
| 1. Hospital executives respect all staff opinions | 1 | 2 | 3 | 4 | 5 |
| 1. Hospital executives has paid attention to clinical pharmacy services | 1 | 2 | 3 | 4 | 5 |
| 1. Hospital executives assigns jobs in accordance with the staff's training expertise | 1 | 2 | 3 | 4 | 5 |
| 1. The Head of the Pharmacy department supports you in work | 1 | 2 | 3 | 4 | 5 |
| 1. The Head of the Pharmacy department has paid attention and invested in building strategies to promote clinical pharmacy activities in the hospital | 1 | 2 | 3 | 4 | 5 |
| 1. Your pharmacy colleagues have good cooperation with you | 1 | 2 | 3 | 4 | 5 |
| 1. You have an affable relationship with your pharmacy colleagues | 1 | 2 | 3 | 4 | 5 |
| 1. You have a good professional collaboration with physicians | 1 | 2 | 3 | 4 | 5 |
| 1. You have a good professional collaboration with nurses | 1 | 2 | 3 | 4 | 5 |
| 1. Coworkers support each other in life | 1 | 2 | 3 | 4 | 5 |
| 1. Coworkers are willing to share experiences and support each other in work | 1 | 2 | 3 | 4 | 5 |
| 1. The hospital has policies supporting staff to improve their qualifications (degrees). | 1 | 2 | 3 | 4 | 5 |
| 1. The hospital creates favorable conditions for staff to participate in short-term training courses to improve soft skills (communication skills, informatics,...) | 1 | 2 | 3 | 4 | 5 |
| 1. The hospital organizes continuing medical education courses for members on clinical pharmacy, drug information,.... | 1 | 2 | 3 | 4 | 5 |
| 1. The hospital facilitates employees to participate in courses on hospital management skills (updating pharmaceutical management documents, quality management and patient safety, human resource management, information management. ..) | 1 | 2 | 3 | 4 | 5 |
| 1. You have promotion opportunities during the working process for your effort | 1 | 2 | 3 | 4 | 5 |
| 1. Hospital’s promotion policy is transparent and reasonable | 1 | 2 | 3 | 4 | 5 |
| 1. Hospital publishes all the leadership standards | 1 | 2 | 3 | 4 | 5 |
| 1. Hospital appointes leadership position democratically and equally. | 1 | 2 | 3 | 4 | 5 |
| 1. Your salary matches up to your competence and contribution | 1 | 2 | 3 | 4 | 5 |
| 1. The hospital has appropriate policies in distributing benefits to employees | 1 | 2 | 3 | 4 | 5 |
| 1. Occupational allowance and hazardous allowance matches the dedication | 1 | 2 | 3 | 4 | 5 |
| 1. Bonus and extra income matches the dedication | 1 | 2 | 3 | 4 | 5 |
| 1. Bonus and extra income are distributed fairly, which encourages employees to work harder | 1 | 2 | 3 | 4 | 5 |
| 1. The hospital's internal regulations and rules are clear, realistic and public | 1 | 2 | 3 | 4 | 5 |
| 1. The working environment in hospital departments is democratic. | 1 | 2 | 3 | 4 | 5 |
| 1. The internal spending regulations are fair, reasonable and public | 1 | 2 | 3 | 4 | 5 |
| 1. Hospital ensures the full payment of social insurance, health insurance, periodic health check, other forms of sickness and maternity support | 1 | 2 | 3 | 4 | 5 |
| 1. You are offered vacations regularly | 1 | 2 | 3 | 4 | 5 |
| 1. Hospital organizes sports competitions and arts programs for employees regularly | 1 | 2 | 3 | 4 | 5 |
| 1. The hospital union is active | 1 | 2 | 3 | 4 | 5 |
| 1. Your working is equipped with sufficient sources of clinical pharmacy-related materials | 1 | 2 | 3 | 4 | 5 |
| 1. You are satisfied with the facilities at your workplace (The office is spacious, clean, and airy) | 1 | 2 | 3 | 4 | 5 |
| 1. The hospital has enough equipment for your professional work. (Office equipments, desks and chair,... are adequate; old and outdated equipments will be replaced promptly) | 1 | 2 | 3 | 4 | 5 |
| 1. The learning environment (library, reading room, information search, internet access) creates conditions for you to update your knowledge and to improve your qualifications | 1 | 2 | 3 | 4 | 5 |
| 1. You are equipped with adequate and new protective gears (clothing, mask, gloves,...) and use is unrestricted. | 1 | 2 | 3 | 4 | 5 |
| 1. You are having a safe working environment | 1 | 2 | 3 | 4 | 5 |
| 1. The hospital ensures security and order for you to fully focus on working | 1 | 2 | 3 | 4 | 5 |
| 1. Patients and their families have a respectful and collaborative attitude with you during treatment process | 1 | 2 | 3 | 4 | 5 |
| 1. There are duty rooms for you | 1 | 2 | 3 | 4 | 5 |
| 1. Your duty time is arranged reasonably | 1 | 2 | 3 | 4 | 5 |
| 1. Your current job is in accordance with the expertise in which you are trained | 1 | 2 | 3 | 4 | 5 |
| 1. You have the opportunity to develop your capacity at work | 1 | 2 | 3 | 4 | 5 |
| 1. The workload is assigned appropriately | 1 | 2 | 3 | 4 | 5 |
| 1. Your current job is interesting | 1 | 2 | 3 | 4 | 5 |
| 1. The specialized job meets your personal aspirations | 1 | 2 | 3 | 4 | 5 |
| 1. Overall satisfaction level about the hospital | 1 | 2 | 3 | 4 | 5 |
| 1. Self-assessment of the level of workload completion at the hospital | 1 | 2 | 3 | 4 | 5 |
| 1. Overall satisfaction level on your current job | 1 | 2 | 3 | 4 | 5 |
